# Brazilian Front-of-Package Labeling: A Product Compliance Analysis 12 Months after Implementation of Regulations

**DOI:** 10.3390/nu16030343

**Published:** 2024-01-24

**Authors:** Marcos Vinícius Garcia Senda, António Raposo, Edite Teixeira-Lemos, Cláudia Chaves, Hmidan A. Alturki, Zayed D. Alsharari, Bernardo Romão

**Affiliations:** 1 IESB University Center, Instituto de Educação Superior de Brasilia, Brasília 70200-730, Brazil; marcos.senda@iesb.edu.com; 2 CBIOS (Research Center for Biosciences and Health Technologies), Universidade Lusófona de Humanidades e Tecnologias, Campo Grande 376, 1749-024 Lisboa, Portugal; 3 CERNAS Research Centre, Polytechnic University of Viseu, 3504-510 Viseu, Portugal; etlemos3@gmail.com; 4 ESSV, Centre for Studies in Education and Innovation (CI&DEI), Polytechnic University of Viseu, 3504-510 Viseu, Portugal; claudiachaves21@gmail.com; 5 King Abdulaziz City for Science & Technology, Wellness and Preventive Medicine Institute—Health Sector, Riyadh 11442, Saudi Arabia; halturki@kacst.edu.sa; 6 Department of Clinical Nutrition, Faculty of Applied Medical Sciences, University of Tabuk, P.O. Box 741, Tabuk 71491, Saudi Arabia; zalsharari@ut.edu.sa; 7 Faculty of Health Sciences, Department of Nutrition, University of Brasília, Brasília 70910-900, Brazil

**Keywords:** Brazil, front-of-package labeling, nutrition, public health, ultra-processed foods

## Abstract

This study investigated the presence of front-of-package labeling on food products in major retailers in Brazil after its implementation in 2022. Carried out from May to October 2023, we analyzed 2145 products of brands present in five Brazilian states. A total of 541 products presented front-of-package warnings. The categories varied in their adherence to front-of-package labeling, highlighting a prevalence of combined warnings, such as high in added sugar and high in saturated fat on sweet biscuits and chocolates. Sausages showed a high prevalence of high in sodium and high in saturated fat warnings. Beverages stood out as high in added sugar, while fats, dairy products, frozen preparations, seasonings, and sauces presented diversity in the warnings. Other products, such as panettone, showed a high presence of alerts. The study demonstrated the widespread presence of front-of-package labeling on ultra-processed products highly consumed by the Brazilian population. Considering the alarming presence of these foods in the Brazilian diet, it is concluded that front-of-package nutrition labeling is crucial to inform and raise awareness among consumers, allowing healthier choices and potentially contributing to a reduction in chronic diseases and the costs associated with treatment in the health system.

## 1. Introduction

Mandatory food labeling is recognized worldwide as a public health strategy for promoting adequate nutrition, as well as mitigating excess weight and chronic non-communicable diseases (NCDs) [1,2]. In the European Union (EU), for example, legislation regarding food labeling has been pivotal in promoting healthier dietary choices, as food labels provide clear and accurate information regarding nutritional content, including energy value, fat, saturated fat, carbohydrates, sugars, protein, and salt [3].

In Brazil, since 1999, the first version of the National Food and Nutrition Policy (PNAN) has emphasized the need for nutrition labeling to be mandatory, constituting a measure to prevent health problems and to provide information regarding food’s nutritional composition [4]. Considering these guidelines, the Brazilian National Health Surveillance Agency (ANVISA) decided to publish, in 2000, the first resolution that established this obligation for packaged foods; however, its effective implementation only occurred in the period between 2003 and 2006 due to the need for adaptions in the food productive sector [5,6].

However, after years of mandatory nutrition labeling, ANVISA found practical limitations in the legislation [7]. The requirements for describing portion sizes and their respective nutritional values were described at intervals, resulting in heterogeneity within products of the same category, thus allowing the manipulation of portion sizes to present nutritional values, further complicating the comparison between similar products from different manufacturers [7,8,9].

Also, there were inconsistencies between the rules for nutrition tables and nutrition claims [8]. As a voluntary declaration, nutrition claims were conveyed in an ostensible way, using more friendly language than in nutrition tables and highlighting only positive aspects, without necessarily a direct relationship with the overall quality of the food [7,8,9].

Therefore, the past model could influence food choices and have misleading potential in consumer understanding, as it overestimates the positive nutritional properties in foods with an unbalanced general nutritional composition [5,8,9].

To address these issues, the concept of front-of-package labeling emerged, characterized by the use of simple, easy-to-understand symbols to communicate the key health-related characteristics of foods. Drawing insights from international experiences in over 30 countries, including EU member states, regulatory agencies highlighted the effectiveness of semi-interpretive models developed by governments with mandatory declarations [10].

Thus, after extensive discussion with the public, the Agency decided to publish the Collegiate Board Resolution, RDC No. 429/2020, determining that front-of-package labeling be mandatory on foods whose amounts of additional sugars, saturated fats, or sodium are equal or higher than the limits defined in Normative Instruction (IN) No. 75/2020 [11,12]. In this sense, the aim of this study is to explore compliance with the regulations among major brands for different products in the country.

## 2. Materials and Methods

A quantitative study was carried out on products that adopted the new front-of-package nutrition labeling during the period between May and October 2023. This research was conducted in three distinct phases: the selection of establishments, the selection of products, and data collection and classification.

### 2.1. Selection of Establishments

Initially, the criteria for inclusion of establishments whose goods would be subject to evaluation were defined following studies regarding food label analysis conducted in Brazil [13,14]. In order to ensure greater representation in the analysis, we chose to select hypermarkets present in five different regions of Brazil: north, northeast, center-west, south, and southeast. The specific choice of hypermarkets was due to the fact that they commonly have larger stocks compared to traditional markets. Therefore, the presence of the brand or its respective economic group in at least one state in the respective geographic region was investigated.

### 2.2. Selection of Products

With the markets selected, criteria for the inclusion and exclusion of items in the quantitative survey under analysis were defined. Therefore, in the physical establishments and in e-commerce, an assessment was carried out by food category to identify those that already had front-of-package labeling (FOP). If at least one brand in a given segment displayed FOP, that category and its respective products were incorporated into the scope of the analysis.

However, even if the products were within the scope of Resolution No. 429/2020, if no brand had implemented said legislation, they were excluded from the survey [11].

### 2.3. Data Collection and Classification

After the determination of the included categories in each establishment, the data collection and classification process began. First, the total count of products by type of food was carried out. From this count, the items were separated and classified into different groups according to the definitions of the health regulations in question, such as high in sodium (HS), high in saturated fat (HSF), high in added sugar (HAS), as well as their combinations (HS + HSF, HS + HAS, and HSF + HAS), and products without front-of-package labeling [11].

According to the law, solid foods that present with more than 600 mg/100 g of sodium and liquid foods with more than 300 mg/100 g of sodium are considered high in sodium. Products where the concentration of saturated fat is above 6 g/100 g for solid foods and 3 g/100 g for liquid preparations are high in saturated fat. As for added sugar, concentrations above 15 g/100 g in solid foods and 7.5 g/100 g in liquids are considered high in added sugars [11].

The products were finally categorized into the following groups: biscuits and snacks; sweets and chocolates; sausages and processed meats; drinks; fats; dairy products; frozen ready-made preparations; ready-made seasonings; sauces; and others. The frequencies of foods with front-of-package warnings were then expressed in absolute frequencies with their respective percentages.

## 3. Results

A total of three hypermarket brands present in all five regions of Brazil were analyzed, and 2145 products were included in this evaluation. Of these, the largest quantity was biscuits and snacks (*n* = 651; 30.35%), followed by sweets and chocolates (*n* = 592; 27.60%), and sausages and processed meats (*n* = 259; 12.07%). With smaller amounts, 4.76% (*n* = 102) were classified as beverages, 4.34% (*n* = 93) as fats, 4.10% (*n* = 88) as dairy products, 3.31% (*n* = 71) as frozen ready-made preparations, 3.26% (*n* = 70) as ready-made seasonings, 2.14% (*n* = 46) as sauces, and 8.07% (*n* = 173) as others.

Regarding the presence of front-of-package warnings, a total of 541 products presented a warning, with 15% (*n* = 86) HS, 13% (*n* = 70) HSF, 23.47% (*n* = 127) HAS, 31.43% (*n* = 170) HSF + HAS, 15.71% (*n* = 85) HS + HSF, and 0.5% (*n* = 3) HAS + HS. The remaining products did not meet the criteria for front-of-package labeling.

**Figure 1:**
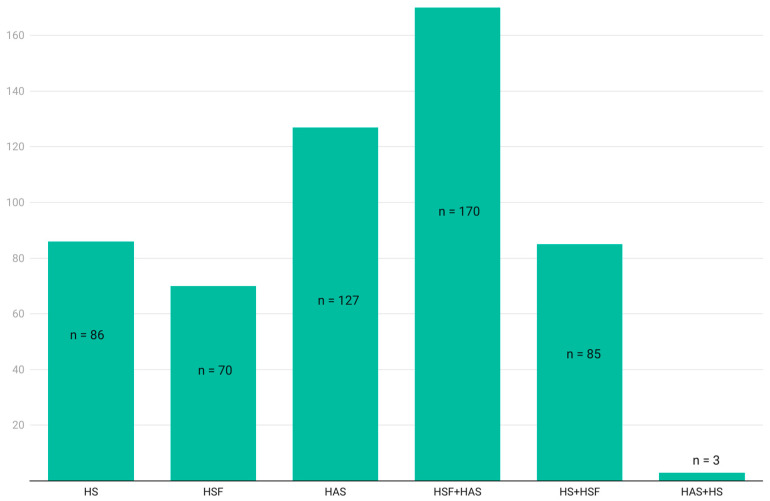
Proportion of products with front-of-package warnings. On the y-axis, number of products; on the x-axis, front-of-package warnings present. HS: high in sodium; HSF: high in saturated fat; HAS: high in added sugar; HSF + HAS: high in saturated fat and high in added sugar; HS + HSF: high in sodium and high in saturated fat; HAS + HS: high in added sugar and high in sodium.

Table 1 displays the detailed results by product category, including the totals analyzed, along with the corresponding numerical amounts and percentages for each type of front-of-package nutrition labeling, as well as for its absence. Figure 1 presents the proportion of products with different front-of-package warnings.

Considering sweet biscuits, the percentages of the HSF + HAS warning varied between 20% and 30% for filled biscuits, wafer biscuits, and wafer biscuits with fillings and toppings. In the case of cookies, this percentage was reduced to 16%, and for cookies without filling, just 4.6%. As for the isolated warning of high added sugar, it was only observed in sweet biscuits with and without filling, representing a percentage of 15%. Like sweets, salty crackers also had warnings on the front, with the highest percentage of 14.8% referring to the high-sodium label.

Within the snack categories, straw potatoes registered a percentage of 44% in high saturated fat, while a comparable percentage (40%) was observed for tapioca biscuits with the HS + HSF warning. In snack foods, the collection of which was the second-most representative in the research, the highest percentage was just 8.4%, associated with the high-sodium (HS) warning.

In the context of sweets and chocolates, the predominant warning was high in added sugar. It was noted, for this specific group, that reduced sampling in some categories resulted in important disparities, with high percentages of the presence of front-of-package labeling, as in the case of sweet popcorn, sweet peanuts, and ice cream mix, in contrast to very low percentages, as in the case of chocolate milk and dulce de leche.

On the other hand, in categories with a more significant number of samples, such as candies and cake mix, the percentage observed for the HAS warning was around 20%.

The cocoa powder category was the only one to display the isolated warning of high in saturated fat, and the chocolate bar and bonbons category, whose quantity was the largest among all the products evaluated in the research, presented only one type of warning (HSF + HAS), at a percentage of 26%.

For sausages and processed meats, the high-sodium and HS + HSF warnings were the most prevalent. As for the first, the percentage with the highest presence was 44% in the cooked ham category, followed by raw ham, which reached 43%. As for the HS + HSF warning, the smoked sausage, bacon, and salami categories recorded the highest percentages, with similar values close to 45%. Still, within this group, raw sausages stood out as the most representative category, showing percentages of approximately 28% for HS labeling, 15% for HS + HSF, and 1.5% for HSF.

With regard to drinks, the only warning observed was high in added sugar, with respective percentages of 8%, 19%, and 29% for energy drinks, soft drinks, and juice syrups.

In fats, butter and margarine stand out as the most representative categories, with the high-saturated-fat warning being more prevalent in margarine (25%), while the HS + HSF warning was exclusively identified in butter (1.7%).

For dairy products, the only exception is related to condensed milk, which displays the warning of high in added sugar instead of high in saturated fat. In the other categories, a wide variation was observed, with percentages ranging from 15% (cottage cheese) to 50% (whipped cream) for the HSF warning.

When analyzing the groups of frozen ready-made preparations and ready-made seasonings, the results focus on the prevalence of high-sodium labeling. Among them, the very low percentage observed for frozen pizzas stands out (2%), while ready-made seasonings show a similar percentage, varying between 22% and 27%.

In the case of sauces, it is worth highlighting that it was the only group of products to present the associated labeling of high in added sugar and high in sodium, with percentages between 8% and 14%.

Finally, in the categories of products classified as other, two extremes stand out. The powdered soup category showed a very low presence of front-of-package labeling, with only 3.8% of 50 products for the high-sodium warning, while the panettone category displayed almost 80% of products with warnings, roughly divided into 56% for HSF + HAS and 22% for HAS.

## 4. Discussion

The growing amount of evidence indicating that unbalanced nutrition represents the main modifiable risk factor for the increased prevalence of overweight and chronic non-communicable diseases (NCDs), with negative impacts, including on the global economy, has motivated several countries to seek solutions more effective in communicating food nutritional information [15,16,17,18]. In addition to strategies focused on health promotion in primary health care, nutrition labeling is a public health measure to better encourage decisions by purchasing healthier products [12,19,20].

Historically, one of the first countries to adopt a front-of-package nutrition labeling model was Sweden in 1989. As a government initiative and of voluntary adoption, the country approved the so-called “keyhole”, a lock symbol that identified healthier options within different categories of food products [21,22].

Due to its effectiveness, other countries also began to adopt this tool, such as Denmark and Norway in 2009 and Iceland and Lithuania in 2014 [21,23]. Another important and well-known model is the nutritional “traffic light”, which uses colors to help understand the level of each nutrient in food (high, medium, or low). The first country to adopt it was the United Kingdom in 2006, followed by similar adoptions by South Korea in 2011, Ecuador in 2013, and Iran in 2015 [24,25,26].

In 2017, the Chilean government published a report on the effectiveness of the regulation of front-label warnings carried out in the country in 2015 [27]. This document highlights that 92.9% of consumers claimed to understand the information presented. Furthermore, 48.1% reported making comparisons between foods that had nutrition labeling on the front, and 79.1% said that this information impacted their food choices [27].

After this broad review of international experiences and the analysis of possible impacts on the production sector, the Brazilian National Health Regulatory Agency (ANVISA) decided to publish Resolution of the Collegiate Board of Directors RDC No. 429/2020 on 9 October 2020 [11]. As established in Article 51, the standard came into effect after 24 months of publication, that is, on 9 October 2022 [11].

The consumption of added sugars is associated with a greater risk of excess weight, tooth decay, diabetes, and cardiovascular disease [28,29]. It is recommended that daily intake does not exceed 10% of the total energy value (TEV), equivalent to 50 g/daily, based on a caloric intake of 2000 kcal [30]. Saturated fats and sodium, in turn, contribute to an increased risk of cardiovascular diseases, which represent the main cause of deaths and hospital admissions in Brazil [31].

According to the guidelines of the World Health Organization (WHO), to prevent such diseases in a similar way to added sugars, it is recommended that the daily intake of saturated fats should not exceed 10% of the TEV [32], while sodium intake should be kept below 2 g [33].

The last survey conducted in Brazil showed the average daily sugar consumption by the Brazilian population reached 109.9 g per day [34]. Notably, 61% of the population exceeds the WHO recommended amounts [34]. It is also noteworthy that adolescents have an average daily sugar intake that is 18% to 30% higher than that of adults and the elderly, respectively, mainly due to their greater consumption of sugary drinks [34]. Regarding sodium, the data indicate an average daily consumption of 3.2 g, with 70% of the population exceeding the maximum value recommended by the WHO [34].

In the products examined in this research, the wide prevalence of ultra-processed products is evident, following the tendency found in the global food system [35]. Recent and representative data indicate that ultra-processed foods correspond to approximately 20% of the total energy consumed daily by the Brazilian population [18], which is significant. For the age group 10 to 19 years old, the adjusted percentage is even higher, reaching almost 30% [18]. Among the most consumed products are margarine, savory biscuits and snacks, sweet biscuits, cold cuts and processed meats, chocolates and industrialized desserts, soft drinks, ready-made dishes, and ready-made sauces, among others [18].

An analysis of ultra-processed foods in Brazil showed an estimate of approximately 57000 premature deaths per year associated with their high consumption, even exceeding the number of homicide victims in the country [36]. The evaluation method used considers the growing body of research that increasingly highlights the relationship between the consumption of these foods, weight gain, and an increased risk of various diseases, including diabetes, cardiovascular diseases, and cancer [36].

There is also evidence of an increased risk of cardiovascular disease, stroke, and mortality from ischemic heart disease in places where there is a greater supply of ultra-processed foods [37].

Evaluating the results of this study, it is noted that all the categories of products mentioned above and of high consumption by the Brazilian population display front-of-package nutrition labeling warnings. The biscuits and snacks groups (20–45 g/daily), as well as sweets and chocolates (18–38 g/daily), stand out in the analysis, representing 58% of the products evaluated [34]. Also, the prominent presence of combined warnings indicating high-saturated-fat content together with high-sodium content or high-added-sugar content in these groups is noted.

On the other hand, it is important to highlight the presence of warnings on foods that do not fall into the ultra-processed category, such as butter, cocoa powder, olives, and fresh pasta [38,39]. Even though they do not contain additives, the natural presence of saturated fat or the addition of salt by the industry for conservation, for example, results in significant quantities of these ingredients in its composition [40,41,42]. Therefore, the consumption of these products must also be considered for a balanced diet.

Different hypotheses are associated with the low percentages of the presence of front-of-packaging labeling in some categories. The first is related to the effort on the part of the food industry to reformulate the components of products so that they do not have to use the mandatory front-of-package labeling. For example, the total or partial replacement of sugar (sucrose) with synthetic sweeteners such as aspartame and sucralose or even natural sweeteners such as stevia or xylitol is a worldwide-adopted strategy [43,44].

Furthermore, given the time needed to adapt the labeling of products already available in the markets, it is possible that at the time of data collection, such labels had not yet been replaced, especially considering the long shelf life of ultra-processed products.

Among the various objectives of front-of-package nutrition labeling, the main one is related to consumer empowerment, allowing informed food choices and respecting freedom of choice and individual interests [7,12]. This tool makes it possible to improve choices for consumers who are more familiar with the topic, as well as help those who have difficulty understanding such information [7,12].

With this change, it is expected that consumers will more easily understand the nutritional characteristics of foods that directly impact their health and will even be able to make nutritional comparisons between products in the same or different categories. Another benefit is the possibility of companies reformulating products so as not to display front-label warnings, resulting in an increase in the availability of healthier alternatives to consumers.

These potential changes characterize front-of-package nutrition labeling as an important public health instrument, which can contribute to improving the population’s quality of life, help reduce the prevalence of chronic illnesses, and thus reduce the direct and indirect costs associated with individual treatments, especially for Brazil’s Unified Health System (SUS).

As a strength of the study, it is important to highlight that it is the first evaluation carried out after the new labeling legislation in Brazil came into force; however, considering the 20% variation allowed between the real nutritional value and the value declared on the label, to present more robust evidence, laboratory analysis of these foods using validated methods is suggested for future studies. Also, as all the presented data are based on absolute frequencies, further studies may apply more robust statistics to better elucidate the subject.

## 5. Conclusions

In essence, while the study underscores the prominence of front-of-package nutrition labeling warnings, it also highlights the complexities associated with diverse product categorizations, formulation modifications by industries, and temporal discrepancies in label adaptation. These findings emphasize the importance of the continual monitoring and effective implementation of nutrition labeling regulations to empower consumers by providing transparent information and aiding them in making informed dietary choices aligned with their overall health and well-being.

## Figures and Tables

**Table 1:** Quantities and percentages of included product categories divided by type of front-of-package labeling and its absence.

	Total	HS		HSF		HAS		HSF + HAS		HS + HSF		HAS + HS		Without Warning	
Category of Product	*n*	*n*	%	*n*	%	*n*	%	*n*	%	*n*	%	*n*	%	*n*	%
	Biscuits and Snacks														
Filled Sweet Biscuits	135	-	-	-	-	21	15.6	30	22.2	-	-	-	-	84	62.2
Sweet Biscuits without Filling	108	-	-	-	-	16	14.8	5	4.6	-	-	-	-	87	80.6
Wafer with Filling and Topping	21	-	-	-	-	-	-	6	28.6	-	-	-	-	15	71.4
Wafer without Filling	51	-	-	-	-	-	-	14	27.5	-	-	-	-	37	72.5
Cookies	31	-	-	-	-	-	-	5	16.1	-	-	-	-	26	83.9
Salty Crackers	101	15	14.8	3	3.0	-	-	-	-	3	3	-	-	80	79.2
Appetizer	155	13	8.4	6	3.9	-	-	-	-	6	3.9	-	-	130	83.9
Tapioca Biscuit	5	-	-	-	-	-	-	-	-	2	40	-	-	3	60
Straw Potatoes	25	-	-	11	44	-	-	-	-	-	-	-	-	14	56
Salted Peanuts	19	1	5.3	1	5.3	-	-	-	-	-	-	-	-	17	89.5
	Sweets and Chocolates														
Chocolate Bars	305	-	-	-	-	-	-	81	26.6	-	-	-	-	224	73.4
Chocolate Powder	5	-	-	-	-	2	40	-	-	-	-	-	-	3	60
Cacao Powder	2	-	-	1	50.0	-	-	-	-	-	-	-	-	1	50
Cocoa Powder	10	-	-	-	-	1	10	-	-	-	-	-	-	9	90
Peanut Candy	14	-	-	-	-	5	35.7	-	-	-	-	-	-	9	64.3
Sweet Peanuts	5	-	-	-	-	3	60	-	-	-	-	-	-	2	40
Cake Mixes	75	-	-	-	-	15	20	-	-	-	-	-	-	60	80
Sugar Candies	71	-	-	-	-	14	19.7	-	-	-	-	-	-	57	80.3
Hazelnut Spread	7	-	-	-	-	-	-	2	28.6	-	-	-	-	5	71.4
Ice Cream Topping	5	-	-	-	-	2	40	-	-	-	-	-	-	3	60
Ice Cream Powdered Mixture	4	-	-	-	-	3	75	-	-	-	-	-	-	1	25
Chewing Gum	31	-	-	-	-	12	38.7	-	-	-	-	-	-	19	61.3
Honey Bun	5	-	-	-	-	-	-	1	20.0	-	-	-	-	4	80
Sweet Popcorn	2	-	-	-	-	2	100	-	-	-	-	-	-	0	0
Jam	32	-	-	-	-	3	9.4	-	-	-	-	-	-	29	90.6
Dulce de Leche	6	-	-	-	-	1	16.7	-	-	-	-	-	-	5	83.3
Guava Candy (*Brazilian goiabada*)	9	-	-	-	-	5	55.6	-	-	-	-	-	-	4	44.4
Marshmallow	4	-	-	-	-	1	25	-	-	-	-	-	-	3	75
	Sausages and Processed Meats														
Smoked Sausage	33	-	-	-	-	-	-	-	-	15	45.5	-	-	18	54.5
Raw Sausage	65	18	27.7	1	1.5	-	-	-	-	10	15.4	-	-	36	55.4
Hot Dog Sausage	23	3	13.0	-	-	-	-	-	-	5	21.7	-	-	15	65.2
Bacon	23	-	-	-	-	-	-	-	-	11	47.8	-	-	12	52.2
Salami	38	1	2.6	-	-	-	-	-	-	17	44.7	-	-	20	52.6
Hamburger	33	2	6.1	10	30.3	-	-	-	-	2	6.1	-	-	19	57.6
Canned Ham	3	1	33.3	-	-	-	-	-	-	-	-	-	-	2	66.7
Cooked Ham	9	4	44.4	-	-	-	-	-	-	-	-	-	-	5	55.6
Raw Ham	7	3	42.9	-	-	-	-	-	-	-	-	-	-	4	57.1
Mortadella	23	1	4.3	-	-	-	-	-	-	9	39.1	-	-	13	56.5
	Beverages														
Soft Drinks	58	-	-	-	-	11	19	-	-	-	-	-	-	47	81
Energy Drinks	37	-	-	-	-	3	8.1	-	-	-	-	-	-	34	91.9
Refresher Syrup	7	-	-	-	-	2	28.6	-	-	-	-	-	-	5	71.4
	Fats														
Lard	1	-	-	1	100	-	-	-	-	-	-	-	-	0	0
Butter	60	-	-	10	16.7	-	-	-	-	1	1.7	-	-	49	81.7
Margarine	32	-	-	8	25	-	-	-	-	-	-	-	-	24	75
	Dairy														
Cream Cheese	39	-	-	6	15.4	-	-	-	-	-	-	-	-	33	84.6
Canned Whipped Cream	10	-	-	5	50	-	-	1	10.0	-	-	-	-	4	40
Milk Cream	18	-	-	6	33.3	-	-	-	-	-	-	-	-	12	66.7
Sweet Condensed Milk	21	-	-	-	-	5	23.8	-	-	-	-	-	-	16	76.2
	Frozen Ready-Made Preparations														
Frozen Meatballs	1	-	-	1	100	-	-	-	-	-	-	-	-	0	0
Frozen Sandwich	19	8	42.1	-	-	-	-	-	-	-	-	-	-	11	57.9
Frozen Pizza	51	1	2	-	-	-	-	-	-	2	3.9	-	-	48	94.1
	Ready-Made Seasonings														
Ready-Made Seasoning in Tablets	26	7	26.9	-	-	-	-	-	-	-	-	-	-	19	73.1
Ready-Made Seasoning Powder	32	7	21.9	-	-	-	-	-	-	-	-	-	-	25	78.1
Salad Dressing	12	-	-	-	-	-	-	-	-	2	16.7	-	-	10	83.3
	Sauces														
Mayonnaise	19	1	5.3	-	-	-	-	-	-	-	-	-	-	18	94.7
Ketchup	12	-	-	-	-	-	-	-	-	-	-	1	8.3	11	91.7
Barbecue	7	-	-	-	-	-	-	-	-	-	-	1	14.3	6	85.7
Soy Sauce	8	1	12.5	-	-	-	-	-	-	-	-	1	12.5	6	75
	Others														
Pastry Dough	7	1	14.3	-	-	-	-	-	-	-	-	-	-	6	85.7
Fresh Ravioli Pasta	12	4	33.3	-	-	-	-	-	-	-	-	-	-	8	66.7
Morning Cereal (e.g., Sugared Corn Flakes)	41	-	-	-	-	10	24.4	-	-	-	-	-	-	31	75.6
Olives	16	4	25	-	-	-	-	-	-	-	-	-	-	12	75
Soup Powder	52	2	3.8	-	-	-	-	-	-	-	-	-	-	50	96.2
Panettone	45	-	-	-	-	10	22.2	25	55.6	-	-	-	-	10	22.2

HS: high in sodium; HSF: high in saturated fat; HAS: high in added sugar; HSF + HAS: high in saturated fat and high in added sugar; HS + HSF: high in sodium and high in saturated fat; HAS + HS: high in added sugar and high in sodium.

## Data Availability

Data are contained within the article.
